# A human PCLS model of lung injury and repair for discovery and pharmaceutical research

**DOI:** 10.1186/s12931-025-03314-6

**Published:** 2025-07-05

**Authors:** Esther Bankole, Chun Wai Wong, Sally Kim, Matthew Hind, Charlotte H. Dean

**Affiliations:** 1 National Heart and Lung Institute (NHLI), Imperial College London, London, UK; 2 Current address, School of Cancer and Pharmaceutical Sciences, King’s College London, London, UK; 3 National Institute for Health Research (NIHR) Respiratory Biomedical Research Unit, Royal Brompton & Harefield NHS Foundation Trust, London, UK

**Keywords:** Human tissue, Precision-cut lung slices, PCLS, Lung injury, Tissue repair, *Ex vivo* model, Cell biology, Progenitors, Proliferation

## Abstract

**Background:**

The Acid Injury and Repair (AIR) model is an ex-vivo model of lung injury and repair, that was previously established using mouse precision-cut lung slices (PCLS). The AIR model provides a bridge between the current in -vitro and in-vivo models to study the effects of lung injury in 3D lung tissue slices. Here, we show that the AIR model can be adapted for use in human tissue as a translational model for discovery research and drug screening.

**Methods:**

To generate PCLS, resected human lung tissue was coated with alginate hydrogel to form an artificial pleura. Lung tissue was inflated by point injecting 3% agarose, followed by generation of 450–500 µM thick slices of tissue. An isolated area of each slice was injured by brief application of 0.1 M hydrochloric acid. AIR-PCLS were then washed and cultured for 48 h before immunostaining to assess proliferating cells (Ki67) alveolar type II/progenitor cell markers (HTII, proSP-C), lipofibroblasts (ADRP) and endothelial cells (ERG). Viability of PCLS was assessed by both MTT assay and Live/Dead staining.

**Results:**

We show that levels of proliferation do not change in response to acid injury. However, there is a significant increase in the percentage of proSP-C and HTII positive cells in the injured regions of AIR-PCLS. We also identify non-epithelial cell populations; lipofibroblasts and endothelial cells in human AIR-PCLS, to demonstrate that other repair relevant cell types can be identified and tracked in the human AIR (hAIR model).

**Conclusions:**

The hAIR model is an effective ex-vivo tool to study early mechanisms of lung repair following injury. By establishing an area of injured tissue adjacent to uninjured tissue, this model mimics the heterogenous pattern of lung injury frequently present in lung diseases. The hAIR model will facilitate mechanistic studies of human lung repair and provides a valuable pre-clinical model for drug testing.

**Supplementary Information:**

The online version contains supplementary material available at 10.1186/s12931-025-03314-6.

## Background

There is a paucity of disease modifying treatments for adult lung diseases, many of which involve dysregulated repair. The lack of new treatments is in part due to the need for more complex translational models that adequately reproduce the complexity of the in-vivo lungs. The lungs are comprised of numerous cell types ranging from structural components such as epithelial and endothelial cells to immune cell populations including macrophages and neutrophils [1]. In addition, the lungs contain other important components, such as the extra-cellular matrix (ECM), that are critical for both structure and function [2]. Simple cell culture models lack many critical components of the lungs and consequently do not accurately reflect the in-vivo environment. This has led to the development of 3D models to more accurately represent the environment in vivo [3].

Human/non-animal 3D models (NAMs) have been rapidly adopted because they replace animal use as well as improving the translational pipeline [4] however, many of these NAMs are still relatively simple and in most cases do not accurately represent the multifaceted environment of the in-vivo lungs [5].

Precision-cut lung slices (PCLS) have emerged as versatile tools for pre-clinical lung research [6]. These tissue explants contain all structural and some immune cell types, as well as the ECM, in their native arrangements and ratios. Crucially the in-vivo architecture of the lungs is retained. The use of PCLS in respiratory research has expanded with the realisation that this 3D model can be generated from either animal or human lung tissue as well as from control and diseased lung samples [7, 8]. Moreover, PCLS can be adapted to model key lung behaviour such as breathing or hallmarks of disease such as ECM changes in response to fibrosis [9, 10]. Recent studies have demonstrated that PCLS are an effective model for drug testing, for example PCLS have been employed to identify differential effects of anti-fibrotic drugs Nintedanib and Pirfenidone [6, 7, 11].

The lungs are relatively quiescent during homeostasis and contain populations of stem/progenitor cells that reside at different anatomical positions and which can respond following injury, such as basal cells in the airways and bronchoalveolar stem cells at the bronchoalveolar duct junctions [12]. In the alveoli, alveolar type II epithelial cells (ATII) act as facultative stem cells that are activated following injury to help restore damaged alveolar epithelium [13, 14]. We recently established the AIR model in mouse PCLS. In this model, a spatially restricted injury is briefly applied to a lung slice with hydrochloric acid (HCl). The ensuing alveolar repair and regeneration responses can then be tracked and quantified by labelling relevant cell populations such as ATII cells [15].

Here we report successful translation of the AIR model into human tissue. The human AIR model (hAIR) will facilitate mechanistic studies of early lung repair and regeneration and be a valuable pre-clinical model for drug screening purposes.

## Methods

### Generating human precision-cut lung slices

Adult human lung tissue samples were obtained from the Respiratory Biomedical Unit (BRU) Biobank at the Royal Brompton hospital (ethics reference number 20/SC/0142). Lung tissue was obtained from surgically resected lungs of patients with suspected lung cancer. All tissue used for PCLS was classified as histologically normal.

Lung tissue was immersed and stored in serum-free (SF)-RPMI (Gibco, ThermoFisher Scientific; 61870010) supplemented with 1% v/v penicillin streptomycin (Gibco, ThermoFisher Scientific; 15140122), 0.5% v/v gentamycin (Gibco, ThermoFisher Scientific; 15710064) and 0.1% amphotericin B(Gibco, ThermoFisher Scientific; 15290-018), at 4 °C until PCLS production within 24 h. To seal the resected piece of lung tissue, prior to agarose inflation, an artificial pleura was formed by coating the entire tissue sample in 3% sodium alginate (Sigma; W201502) followed by 3% calcium chloride (Sigma; C3306). When completely coated, the tissue was transferred to a falcon tube containing ice cold buffer (1% Hanks Balanced Salt Solution (HBSS) supplemented with 1% v/v penicillin streptomycin) for 10 min. The lung tissue was then inflated with 3% low melting point agarose (Sigma; A9414) by injecting agarose into multiple different areas around the tissue using a 3 ml syringe and 21G BD Microlance™ 3 needle. Point injection was repeated until the entire piece of tissue was evenly inflated. Lung tissue was returned to ice-cold buffer in-between repeated point injections, to maintain agarose gel and optimal tissue viability. To generate equal sized PCLS for experiments, 8 mm cores of lung tissue were removed from the inflated piece of tissue using 8 mm biopsy punch (Selles Medical; BP-80 F). Tissue cores were mounted onto a Compresstome^®^ VF-510-0Z Tissue Slicer (Precisionary instruments), and slices were cut to a thickness of 450–500 μm. PCLS were transferred to 24-well culture plates containing ice cold SF-RPMI supplemented with 1% v/v penicillin streptomycin and incubated at 37 °C with 5% CO2 overnight. PCLS were washed three times with prewarmed SF-RPMI to remove excess agarose, before beginning experiments.

### hAIR model

**Fig. 1:**
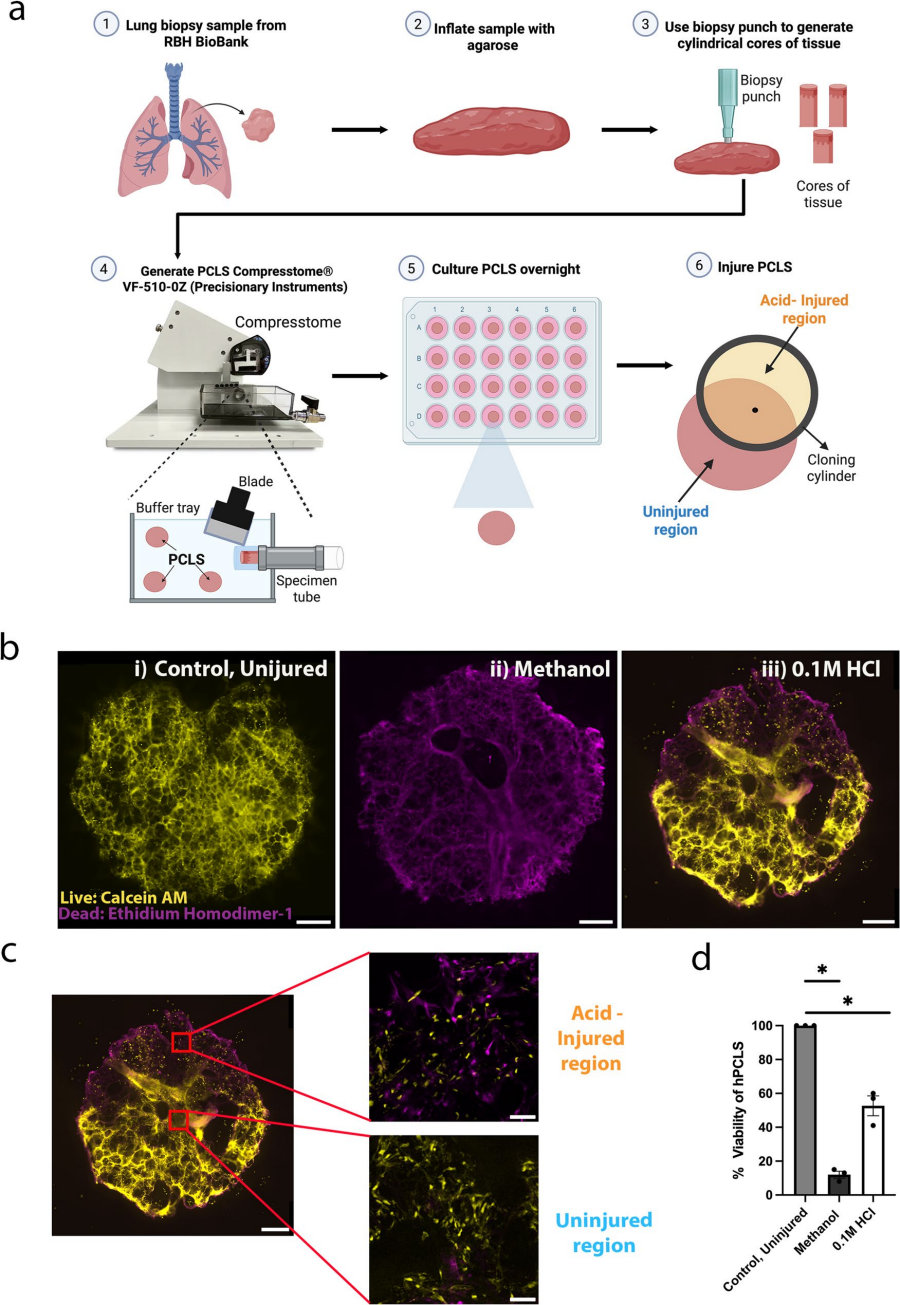
Generation of human PrecisionCut Lung Slices and hAIR model. **(a)** Schematic outlining generation of PCLS using human lung tissue [1–5] and administration of a spatially restricted area of injury using a cloning cylinder [6]. The area of injury is marked with a dot using an alcohol resistant marker pen [6]. **(b)** Widefield whole slice PCLS images taken at 10 × magnification, showing live (Calcein AM, yellow) and dead (Ethidium Homodimer-1, magenta) cells in control, uninjured, methanol and 0.1 M acid-injured slices (*n* = 3) **(c)** Whole slice PCLS image of 0.1 M acid-injured slice with areas of uninjured region and acid-injured region highlighted. Images of uninjured and acid-injured regions were taken at 40x using widefield microscopy. Scale bars: Widefield whole slice = 1000 μm. **(d)** MTT assay demonstrating reduction in PCLS viability with addition of 0.1 M HCl and methanol treatment (negative control) in comparison to control uninjured PCLS (*n* = 3; Kruskal-Wallis test, Dunn’s multiple comparisons test. *p < 0.05)

Using the method described in Kim et al. [15], PCLS were placed in the centre of the well and media was removed. A Pyrex® cloning cylinder (Sigma; CLS31668) was coated at one end with sterile silicone grease and applied to one side of the PCLS to seal the cloning cylinder onto the tissue, creating a barrier between the site of injury and the rest of the tissue. Since the cored hPCLS are circular, an alcohol resistant marker pen was used to mark the side of the slice within the cloning cylinder (Fig. 1a). 500 µl SF-RPMI was added to the area outside the isolated region and the isolated region of the PCLS was injured by adding 100 µl of 0.1 M HCl in 15% w/v Pluronic gel (Sigma; P2443-250G), prepared with SF-RPMI, inside the cloning cylinder, for 1 min. Pluronic gel increased the viscosity of the acid-containing media to help prevent the acid from leaking out of the cloning cylinder sealed with silicone grease. The colour of the phenol-red containing media outside the cylinder was used to monitor if any HCl leaked out i.e. a change from pink to yellow would indicate a leak and the PCLS would then be discarded. After 1 min HCl treatment, the isolated region was washed five times with 200 µl SF-RPMI, ensuring the seal between the two regions was undisturbed. The cloning cylinder was then removed and PCLS were transferred to a 24-well plate containing fresh SF-RPMI and cultured at 37 °C with 5% CO2 for 48 h post injury.

### Viability assays

#### Live/Dead assay

Cell viability was assessed in hAIR model using the LIVE/DEAD^®^ Viability/Cytotoxicity Kit (ThermoFisher Scientific; L3224) following manufacturer’s instructions. PCLS treated with 70% methanol were used as a positive control for dead cells and uninjured/untreated PCLS were used as a positive control for live cells. Calcein AM and Ethidium homodimer-1 (EthD-1) were brought to room temperature (RT) prior to use. PCLS were incubated for with 2 µM Calcien AM and 2 µM EthD-1 in 250 µl warmed HBSS for 30 min at 37 °C. PCLS were washed twice briefly with phosphate buffered saline (PBS) (ThermoFisher Scientific; 28908). Then PCLS were mounted onto glass slides using ProLong^®^ Gold Antifade Mountant (ThermoFisher Scientific; P36930) and were left to set for 30 min at RT and imaged immediately after. Whole slice tiled images were taken at 10 × using a Zeiss Axio Observer widefield microscope.

#### MTT assay

PCLS of equal size were placed into wells of a 48-well plate (1 PCLS per well) in triplicate. To ensure injury/treatment was kept consistent between PCLS for each condition, whole slices were injured/treated. At 48 h post injury/treatment, media was removed from wells and replaced with 250 µl 10% MTT solution (Sigma; M2128) prepared from 5 mg/ml Stock diluted in SF-RPMI, per well. PCLS were incubated for 1 h at 37 °C. MTT was removed, and 250 µl dimethyl sulfoxide (DMSO) was added for 10 min to solubilise formazan crystals that had formed. 200 µl solution was removed from each well and transferred to a 96- well plate. Absorbance (optical density O.D.) was measured at 570 nm and corrected at 690 nm using a SpectraMax iD3 Multi-Mode Microplate Reader (Molecular Devices, USA). Methanol treated slices were used as tissue with no metabolic activity and control, uninjured PCLS were included as a positive control with high metabolic activity.

#### Immunofluorescence of PCLS

PCLS were fixed with 4% Paraformaldehyde (PFA) for 15 min, washed three times with PBS and then permeabilised, by incubating with 0.5% Triton™ X-100 (ThermoFisher Scientific; 28313) on a plate shaker at room temperature (RT) for 30 min. Slices were then washed three times for 5 min in PBS then incubated in blocking buffer PBS-BT (PBS with 1% BSA; Sigma; Cat. No. A7030 and 0.2% Triton X-100) for 1 h to prevent non-specific binding of antibodies. PCLS were incubated with rabbit anti-prosurfactant protein-C (Millipore; Cat. No. AB3786) and rat anti-Ki67 (eBioscience; Cat. No. 14-5698-82; Clone SolA15) antibodies at a concentration of 1:500 or mouse anti-HTII (Terrace Biotech; TB-27AHT2-280) at 1:150, rabbit anti- ADRP (Abcam; ab52356) at 1:200 or rabbit anti-ERG (Abcam; ab92513) at 1:200 in PBS-BT and incubated overnight at 4 °C on a plate shaker. PCLS were washed with PBS three times for 5 min, before incubation with secondary antibodies diluted in PBS-BT for 2 h at RT in the dark on a plate shaker. Secondary antibodies were diluted 1:500 in PBS-BT as follows: Goat anti-rabbit IgG, Alexa Fluor 647 (Invitrogen; A-21244) for proSP-C, ADRP and ERG, goat anti-rat IgG, Alexa Fluor 568 (Invitogen; A-11077) for Ki67, Donkey anti-mouse IgG, Alexa Fluor 647 (Invitrogen; A-31571) for HTII. PCLS were then washed briefly with PBS three times and counterstained with DAPI (ThermoFisher Scientific; D1306) diluted 1:500 in PBS-BT for 15 min at RT. Finally, PCLS were mounted onto glass slides using ProLong^®^ Gold antifade mountant (ThermoFisher Scientific; P36930). PCLS were imaged at Imperial College Facility for Imaging by Light Microscopy (FILM) using a Zeiss Axio Observer inverted widefield microscope with Apotome.

#### Quantification

The percentage of proSP-C^+^, Ki67^+^ and HTII^+^ cells was calculated by manually counting the total positive cells per field of view. Every experiment (biological replicate) contained three technical replicates per experimental group. For each PCLS (technical replicate), three fields of view were imaged from each region of hAIR-PCLS, injured and uninjured, or from control, uninjured PCLS. The number of positive cells was then expressed as a percentage of the total number of cells, DAPI positive nuclei, in the image. DAPI positive nuclei were quantified using an automated ImageJ/Fiji counting macro designed by Steve Rothery (Facility for Imaging by Light Microscopy, Imperial College London) as described in Kim et al. [15]. The percentage of positive cells for each experiment was determined by calculating the mean percent positive cells from the 3 technical replicates. Experiments were repeated at least three times to ensure a minimum of *N* = 3 biological replicates from which a final mean percent positive cells was calculated.

### Statistical analysis

Graphs were produced using GraphPad Prism version 9. Graphs show the mean percentage obtained from biological replicates. Each individual data point on graphs is a single biological replicate (*n* = 1) calculated by determining mean of technical 3 replicates for each experimental group. The variation between biological replicates is also shown on graphs as ± the standard error of the mean (SEM). Biological replicates are denoted ‘n = x’, in corresponding figure legends. Datasets comparing results from a minimum of 3 biological replicates were analysed using a Kruskal-Wallis test and Dunn’s multiple comparisons post-test. Differences were considered statistically significant for p values  <0.05.

## Results

### Establishing a restricted area of acid injury in human PCLS

To generate an area of spatial acid injury in PCLS, a cloning cylinder, coated with silicone grease, was placed onto the PCLS ensuring a tight seal to ensure separation between the area within the cylinder, from the surrounding lung tissue (Fig. 1a). The area inside the cloning cylinder was marked with an alcohol resistant pen so that the ‘injured’ side of the PCLS could be easily identified later in the experiment. The area outside of the cylinder was filled with 500 µl media and 0.1 M HCl mixed with pluronic gel was applied to the isolated region inside the cylinder for 1 min, to injure the tissue. After this, the injured region was washed with media five times to completely remove any residual acid, the cylinder was removed and the injured PCLS was transferred to a 24-well plate with media and cultured at 37 °C for 48 h prior to analysis (Fig. 1a).

To ensure that a spatially restricted region of injury was present, PCLS were stained with a live/dead viability kit to identify calcein positive, live cells and ethidium homodimer-1 positive, dead cells (Fig. 1b). Uninjured slices predominantly contained calcein positive yellow staining, indicating live cells (Fig. 1 bi) whereas methanol treated PCLS (negative control) showed only ethidium homodimer-1 positive, magenta staining, indicating dead cells (Fig. 1bii). Images of whole hAIR-PCLS revealed a clear difference between the injured and uninjured regions (Fig. 1biii). The injured, region contained a high proportion off magenta positive dead cells, in contrast, the uninjured region, contained mostly yellow, calcein positive, live cells (Fig. 1 biii). High magnification images of hAIR-PCLS confirmed that in the uninjured region, almost all cells were alive whereas in the injured region, the majority of cells were dead (Fig. 1c). Notably however, some live (yellow) cells remained in the injured region of hAIR-PCLS (Fig. 1biii).

To quantify the effect of acid injury on PCLS viability, an MTT assay was carried out to compare the metabolic activity of whole PCLS injured with 0.1 M HCl for 1 min to, uninjured whole PCLS and methanol treated whole PCLS. Methanol treated PCLS showed a significantly reduced level of viability in comparison to control, uninjured PCLS (Fig. 1d, *p* < 0.0001). There was also a significant reduction in PCLS viability between control, uninjured PCLS and PCLS injured with 0.1 M HCl (Fig. 1d, *p* = 0.0428). In comparison to methanol treated PCLS, the metabolic activity of PCLS treated with 0.1 M HCl was higher (Fig. 1d), indicating that some live, metabolically active cells remain, even after HCl injury.

### ProSP-C positive cells increase in the injured region, following acid treatment

**Fig. 2:**
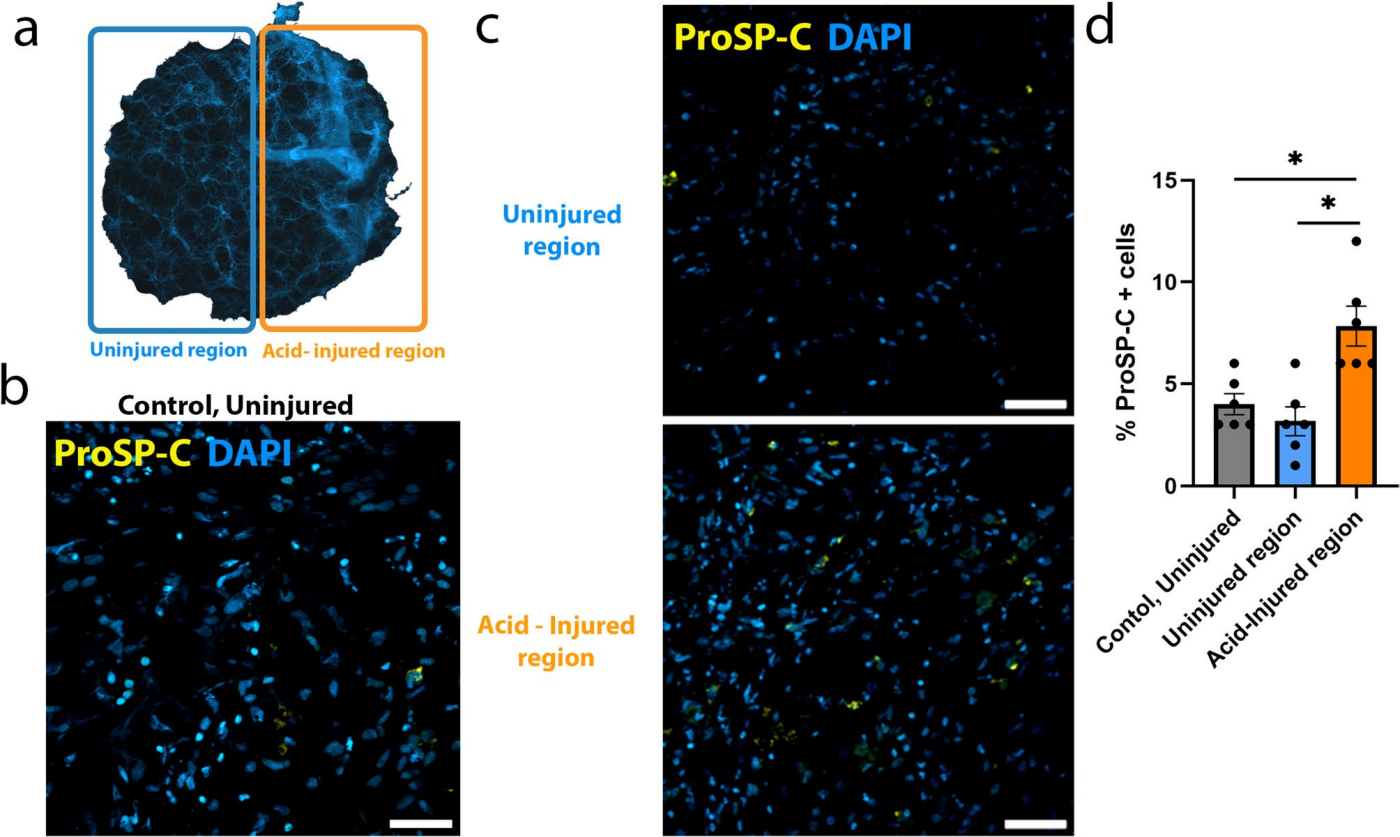
ProSP-C positive cells increase in response to acid-injury. **(a)** Schematic illustrating the uninjured region (blue box) and the acid-injured region (orange box) of hAIR- PCLS. **(b)** Immunofluorescence staining of control, uninjured PCLS with proSP-C (yellow) and DAPI (blue). **(c)** Immunofluorescence staining of uninjured and acid-injured region of hAIR-PCLS with proSP-C (yellow) and DAPI (blue). **(d)** Quantification of proSP-C^+^ cells in control, uninjured PCLS compared to uninjured region (blue bar) and acid-injured region (orange) of hAIR-PCLS 48 h post injury (*n* = 6; Kruskal-Wallis test, Dunn’s multiple comparisons test. *p < 0.05). Widefield images taken at 40 × magnification, scale bars = 50 μm

To visualise and quantify alveolar repair, hAIR-PCLS were cultured for 48 h and then fixed and immunostained with anti-pro-surfactant protein C (ProSP-C), a marker of alveolar type II progenitor cells, that are known to increase following injury to facilitate repair [15]. For each PCLS, the number of proSP-C^+^ cells were quantified in three separate fields of view within the uninjured and injured regions of hAIR-PCLS (Fig. 2). The percentage of these cells was calculated in comparison to the total number of DAPI positive nuclei within each field of view. The average percentage of proSP-C^+^ cells in the uninjured region of hAIR-PCLS was 3%, a similar percentage to that found in control, uninjured PCLS (Fig. 2c, d). In contrast, there was an increased percentage of proSP-C^+^ cells in the injured regions of hAIR-PCLS to an average of 8% (Fig. 2c, d). In agreement with data from mouse AIR-PCLS [15], there was also a significant increase in proSP-C^+^ in the acid-injured region of hAIR-PCLS compared to both the uninjured region of hAIR-PCLS (Fig. 2c, d, *p* = 0.0065) and to control, uninjured PCLS (Fig. 2b-d, *p* = 0.0487). *N* = 6 donors i.e. biological replicates.

### Proliferation does not increase in PCLS following acid injury

**Fig. 3:**
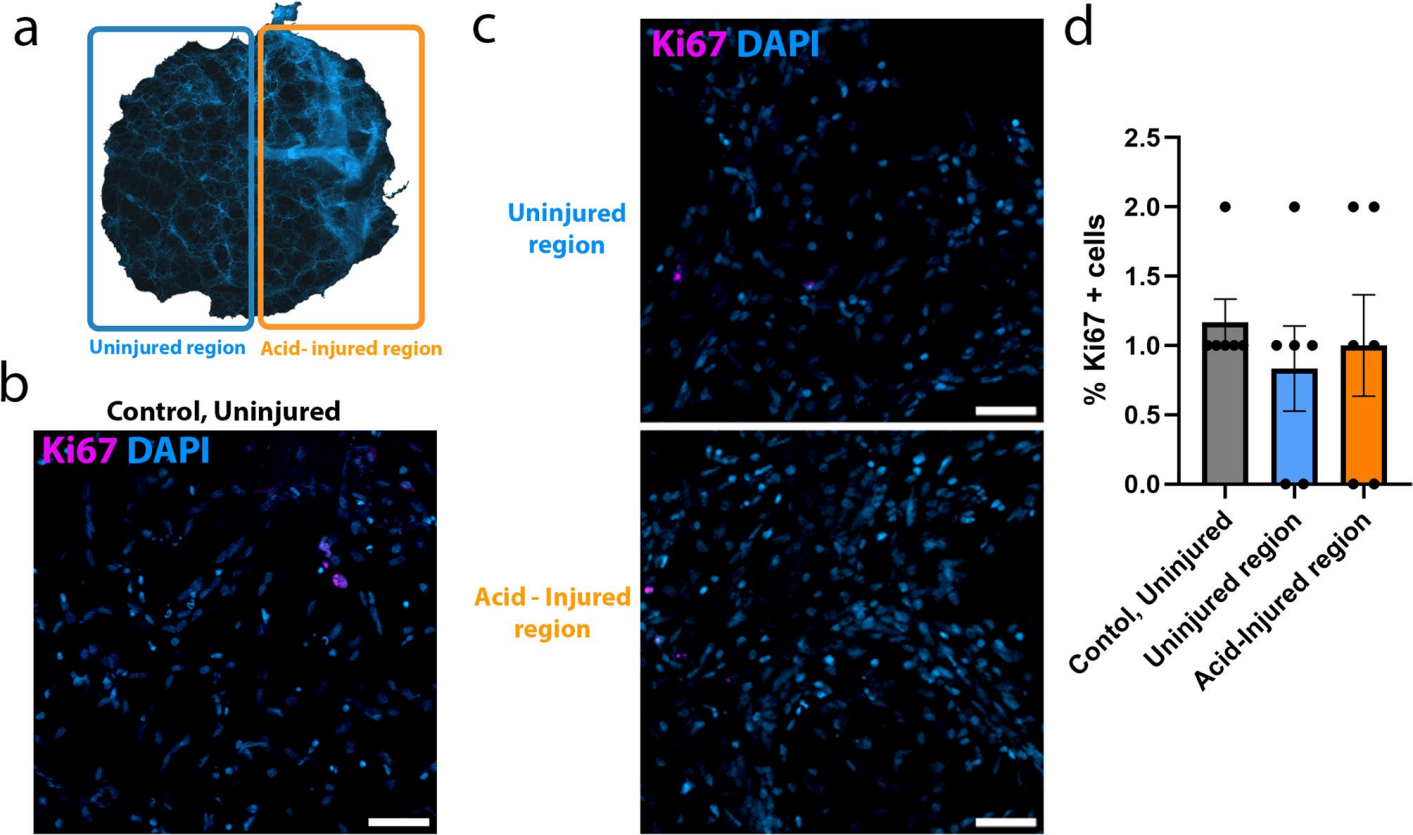
Percentage of proliferating cells does not change in response acid-injury. **(a)** Schematic illustrating the uninjured region (blue box) and the acid-injured region (orange box) of hAIR-PCLS. **(b)** Immunofluorescence staining of control, uninjured PCLS with ki67 (pink) and DAPI (blue). **(c)** Immunofluorescence staining of uninjured and acid-injured region of AIR PCLS with ki67 (pink) and DAPI (blue). **(d)** Quantification of ki67^+^ cells in control, uninjured PCLS compared to uninjured region (blue bar) and acid-injured region (orange bar) of hAIR-PCLS 48 h post injury (*n* = 6; Kruskal-Wallis test, Dunn’s multiple comparisons test. *p < 0.05). Widefield images taken at 40 × magnification, scale bar = 50 μm

Cell proliferation is frequently associated with repair and regeneration. To track changes in proliferation, post acid-injury, hAIR-PCLS were immunostained with anti-Ki67 antibody. As previously described for proSP-C^+^ cells, the percentage of Ki67^+^ cells was quantified in three separate fields of view within control, uninjured PCLS and compared to the percentage in hAIR-PCLS spatially injured with 0.1 M HCl (Fig. 3a). The mean percentage of Ki67^+^ cells in control, uninjured PCLS was 1.2% (Fig. 3b, d). The mean percentage of Ki67^+^ was 0.8% in the uninjured region and 1% in the acid injured region of hAIR-PCLS (Fig. 3c, d), indicating that 48 h after injury, there was no significant difference in proliferation between the injured and uninjured region of hAIR-PCLS (*p* > 0.9999). The mean percentage of Ki67^+^ cells in control, uninjured PCLS (Fig. 3b, d) was also not statistically different from either the injured (*p* > 0.9999) or uninjured region of hAIR-PCLS (*p* = 0.8000), *N* = 6 donors.

### The human specific ATII marker HTII-280 is also increased upon injury in AIR-PCLS

**Fig. 4:**
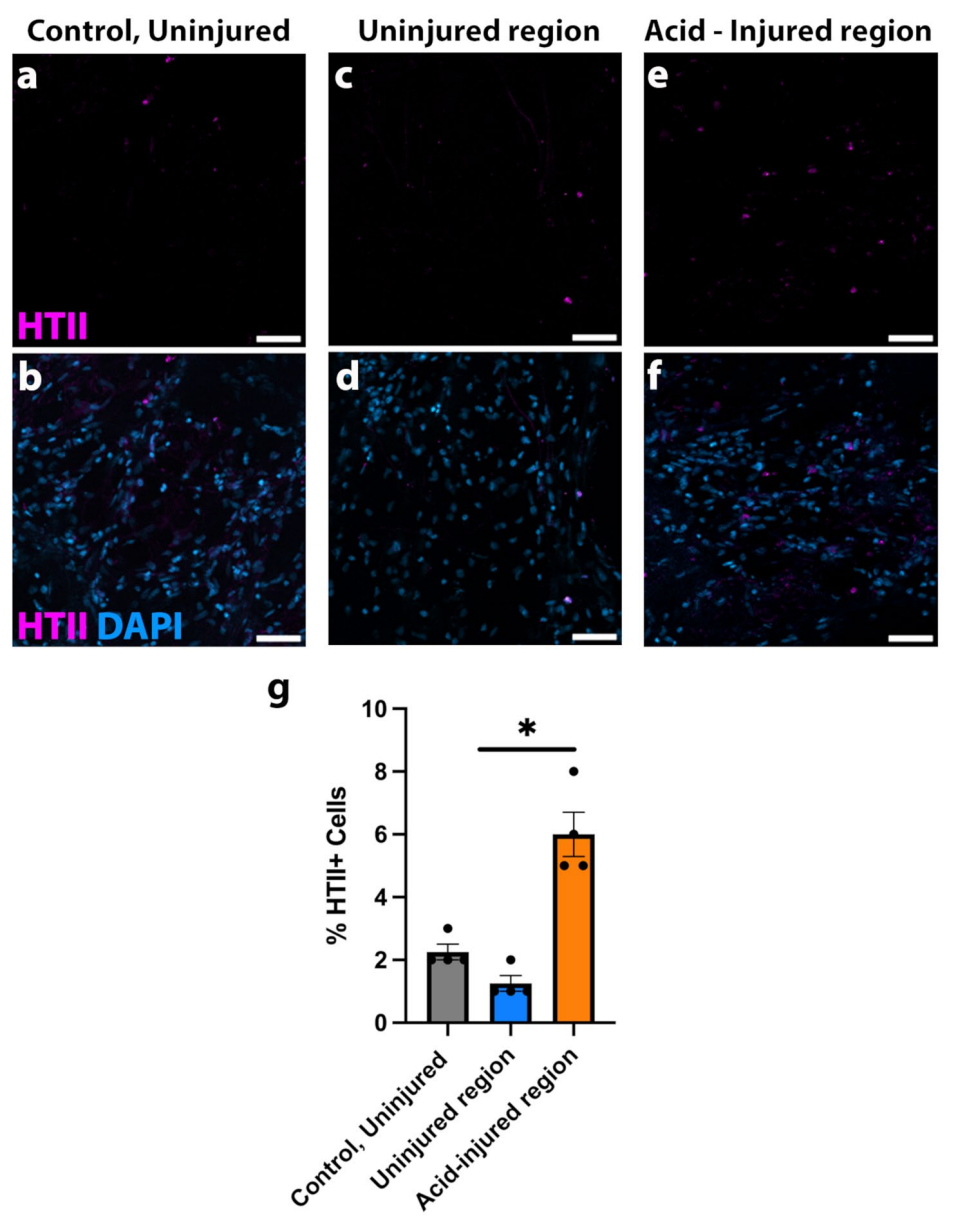
HTII-280 is also increased in the injured region of hAIR-PCLS. **(a-b)** Immunofluorescence staining for HTII (pink) and DAPI (blue) in control, uninjured PCLS. **(c-f)** Immunofluorescence staining for HTII (pink) and DAPI (blue) in the uninjured region **(c, d)** and injured region **(e, f)** of hAIR-PCLS, 48 h post injury **(g)** Quantification of HTII^+^ cells in control, uninjured PCLS compared to uninjured region (blue bar) and acid-injured region (orange bar) of hAIR-PCLS 48 h post injury (*n* = 4; Kruskal-Wallis test, Dunn’s multiple comparisons test. *p < 0.05). Widefield images taken at 40 × magnification, scale bar = 50 μm

HTII is a plasma membrane marker specific to human ATII cells that is frequently used in place of proSP-C in human samples [16]. We immunostained control, uninjured PCLS with an antibody to HTII and as expected, this antibody labelled a population of cuboidal cells in the alveolar region of PCLS (Fig. 4a-f). There was no significant difference between the percentage of HTII^+^ cells in the control, unininjured PCLS (Fig. 4a, b,d) and the uninjured region of hAIR-PCLS (Fig. 4c, d,g *p* = 0.5710). But in agreement with proSP-C data (Fig. 2), there was a significant increase in the percentage of HTII cells in the injured region of hAIR-PCLS to 6% (Fig. 4e, f,g *p* = 0.0064), *N* = 4 donors.

### Lipofibroblasts and endothelial cells can be identified and tracked in hAIR-PCLS

**Fig. 5:**
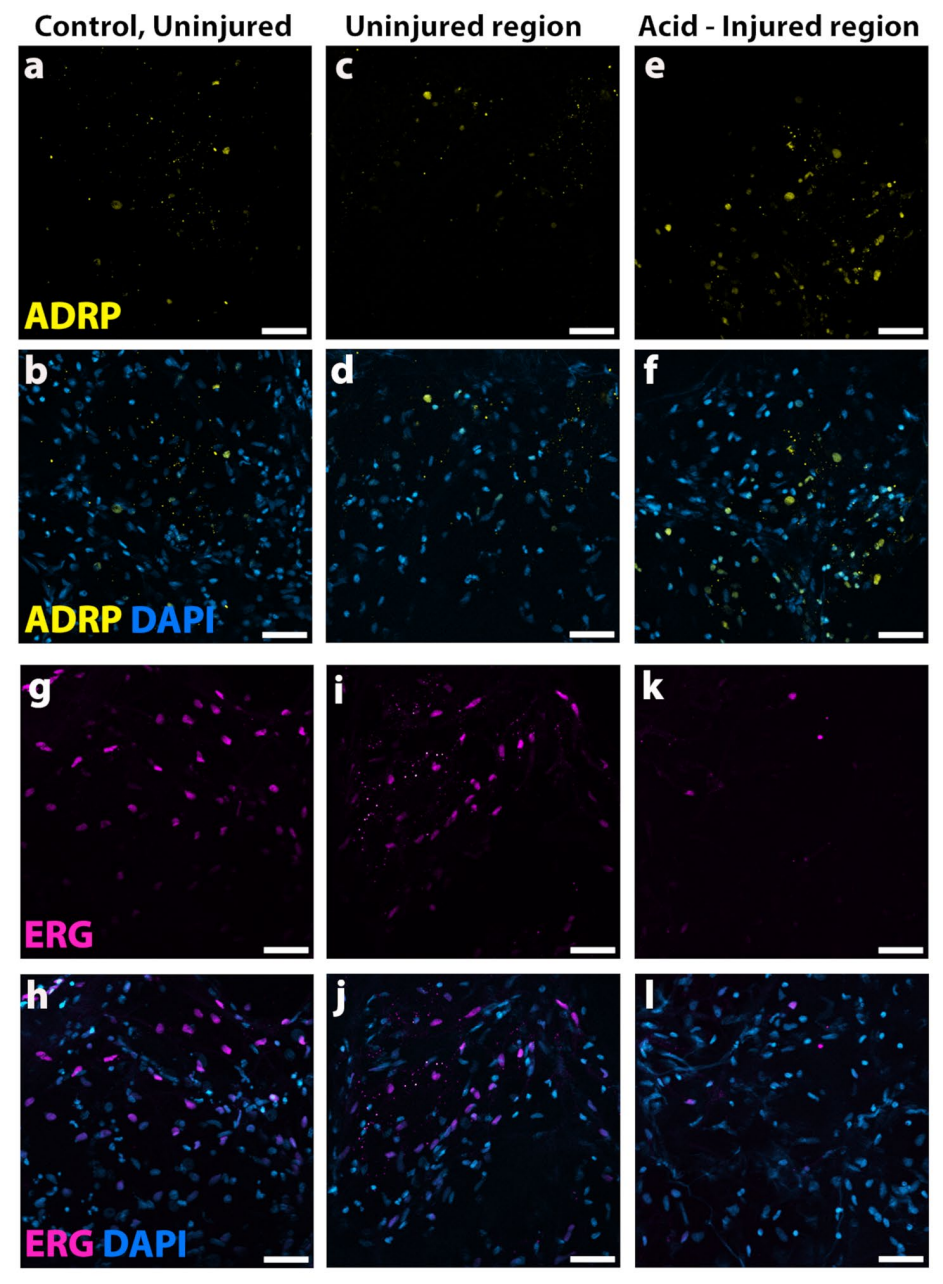
The hAIR model can be used to investigate fibroblast cell populations and endothelial cells. **(a-b)** Immunofluorescence staining for fibroblast cell marker ADRP (yellow) and DAPI (blue) in control, uninjured PCLS. **(c-f)** Immunofluorescence staining for ADRP (yellow) and DAPI (blue) in the uninjured region **(c, d)** and injured region **(e, f)** of hAIR-PCLS, 48 h post injury **(g-h)**. Immunofluorescence staining for endothelial cell marker ERG (pink) and DAPI (blue) in control, uninjured PCLS. **(i-l)** Immunofluorescence staining for ERG (pink) and DAPI (blue) in the uninjured region **(i, j)** and injured region **(k, l)** of hAIR-PCLS, 48 h post injury. Widefield images taken at 40 × magnification, scale bar = 50 μm

In addition to epithelial cells, other cell types and extracellular components also have an important role in lung repair and regeneration. Among these are lipofibroblasts and endothelial cells [17, 18]. We identified both these cell types in control, untreated hPCLS (Fig. 5a, b, c, h). Although we did not conduct quantitative analysis of these two cell types, interestingly there appeared to be more ADRP + cells (Fig. 5e, f) but fewer ERG ^+^ cells (Fig. 5k, l) in the acid-injured region of hAIR-PCLS, compared to either control, uninjured PCLS (Fig. 5a, b, g, h), or the uninjured region of hAIR-PCLS (Fig. 5c, d, I, j).

## Discussion

Here we have established that spatial acid injury of human PCLS can be used as a pre-clinical model to study the lungs’ cellular and molecular response to injury. Immunostaining of ATII progenitor cells with either proSP-C or the human specific marker HTII revealed an increase in progenitor cells within the injured region of PCLS, 48 h after acid treatment. The increase in progenitor cells agrees with previous AIR data from the mouse [19] and with studies of lung repair and regeneration in vivo [14, 20, 21]. In addition, we identified lipofibroblasts and endothelial cells, two other repair relevant cell populations in hPCLS, showing that multiple different cell types and can be tracked in the hAIR model.

This model is particularly well adapted for studies of human lung disease pathogenesis because each hAIR-PCLS contains a region of injured tissue immediately adjacent to an area of healthy tissue, producing a heterogeneous pattern of injury which is frequently present in lung diseases, for example, Idiopathic Pulmonary Fibrosis (IPF) and Chronic Obstructive Pulmonary Disease (COPD) [22]. Therefore, the hAIR model enables detailed investigation of cross-talk between the damaged region and healthy cells/tissue components in the uninjured region at the cellular level. Cross-talk between healthy and damaged tissue is likely to be a critical driver of repair in vivo, but to date this has been difficult to address in human lung models, other than by spatial transcriptomics [23].

Interestingly, we did not observe any change in proliferation following acid treatment; there could be several reasons for this. Whilst proliferation is often a key aspect of repair and regeneration, there are other equally important processes through which epithelial progenitor cells act to restore denuded areas such as cytoskeletal re-organisation, migration and spreading [24]. It is also possible that we could have missed the time window when proliferation occurs, since we only assessed proliferation 48 h after acid treatment. However, in the mouse AIR model, we did not find any significant changes in proliferation at the earlier timepoint of 24 h post-injury. Interestingly, in the mouse AIR model, we identified increased proliferation in the uninjured region of PCLS at 48 h, suggesting that the adjacent healthy tissue might be responding to the injury [19], but this was not observed in human AIR-PCLS. Other studies have shown that there is a considerable amount of cell plasticity in the lung parenchyma, particularly following injury [25, 26]. Therefore, it may be that trans-differentiation contributes to the increase in progenitor cells within the injured region of hAIR-PCLS.

In this study we chose acid to injure the lung tissue. Acid is a clinically relevant driver of lung injury caused by gastric acid aspiration [27] and an important cause of exacerbations in several prominent lung diseases such as COPD and IPF [28, 29]. Additionally, Hydrochloric acid is readily available in most laboratories and produces a non-infectious, sterile injury that is highly reproducible between experiments. In future, other mediators of injury such as bleomycin, elastin or TGF-β could be used instead of acid, depending on the specific research question [10, 30]. We concentrated on analysing cell populations in the hAIR-PCLS, however the ECM plays an equally important role in repair and regeneration of tissue as well as in pathobiology of disease [2]. Previous studies have shown that ECM components can be visualised and quantified in human PCLS and they are modified in response to challenges [9], in future it will be interesting to use the hAIR model to investigate ECM responses.

To date we have focused on using PCLS to model repair and regeneration in the lung parenchyma, but a key advantage of PCLS, is that they contain both alveoli and airways. Therefore, this model could easily be adapted to model the response of airway cells to injury by focusing imaging and quantification on airways.

In comparison to other 3D models, PCLS have the advantage that the cellular composition and extracellular structure is largely intact. They retain all the resident lung cell populations including smooth muscle, epithelial, endothelial cells and fibroblasts. They also contain resident macrophages and enable measurement of cytokines in response to a stimulus such as LPS or *Pseudomonas aeruginosa* [6, 7]. However, as with all models, there are some disadvantages to PCLS. Firstly, their viability declines over time in culture and current consensus is that culture duration should be limited to a maximum of 5–7 days [6]. Secondly, there is no circulation and therefore active recruitment of immune cells cannot take place. Finally, treatments are usually added to the culture media surrounding PCLS and therefore all cells and components of PCLS are exposed, whereas in vivo exposure may be more selective.

## Conclusions

Human PCLS have already been shown to be a versatile model for studying lung biology and drug responses. Here we describe the hAIR model and show that this provides a simple and reproducible pre-clinical model to study mechanisms of repair and regeneration and test potential pro-repair drugs.

## Electronic supplementary material

Below is the link to the electronic supplementary material.

Supplementary Material 1

Supplementary Material 2

## Data Availability

No datasets were generated or analysed during the current study.
