# Supplementary material for: A human PCLS model of lung injury and repair for discovery and pharmaceutical research
Source: Respir Res. 2025 Jul 5;26:237. doi: 10.1186/s12931-025-03314-6 (PMC12228282; doi:10.1186/s12931-025-03314-6)
Supplement: Supplementary file 1 — Supplementary Material 1 [file 12931_2025_3314_MOESM1_ESM.docx]

Table 1: Patient Demographics

| **Gender** | **Age** | **Smoking History** |
| --- | --- | --- |
| Female | 70 | Non- smoker |
| Female | 78 | Ex-smoker |
| Male | 64 | Ex-smoker |
| Male | 58 | Ex-smoker |
| Female | 71 | Ex-smoker |
| Male | 72 | Ex-smoker |
| Female | 76 | Ex-smoker |
| Male | 74 | Non-smoker |
| Male | 58 | Ex-smoker |
| Female | 58 | Ex-smoker |
